# Validation Strategy Dominates Model Choice in Chronological QSAR Evaluation

**DOI:** 10.1021/acs.jcim.6c02796

**Published:** 2026-10-02

**Authors:** Filipa Almendra, Andre O. Falcao

**Affiliations:** † Biosystems and Integrative Sciences Institute (BioISI), Faculdade de Ciencias, 451099 Universidade de Lisboa, Campo Grande, Lisboa 1749-016, Portugal; ‡ Departamento de Informatica, Faculdade de Ciencias, 111161 Universidade de Lisboa, Campo Grande, Lisboa 1749-016, Portugal

## Abstract

Quantitative structure–activity relationship (QSAR) models are commonly evaluated by random cross-validation or random train-test splits, but these retrospective procedures may overestimate performance when validation compounds are structurally or temporally close to the training data. In this study, we examined whether this optimism is primarily explained by structural redundancy, chronological data set evolution, or the choice of machine-learning model. Fourteen public ChEMBL bioactivity data sets were curated as binary classification tasks. Five validation conditions were compared: random validation, chronological validation by publication year, a size-matched randomly thinned control, structural filtering of the random validation set, and structural filtering of the chronological validation set. Model development was deliberately held fixed within the random-derived and year-derived comparisons, so that the effect of validation-set composition could be assessed without simultaneously changing the training cohort. Four standard QSAR model classes—random forest, support vector machine, XGBoost, and a feed-forward neural network—were evaluated under the same training-only model-selection workflow. Random validation produced closely aligned internal and external estimates, with mean MCC values of 0.75 and 0.72, respectively, while random thinning gave essentially unchanged validation performance (MCC 0.73). Structural filtering reduced validation MCC to 0.48 without changing the corresponding training-only CV estimate. Chronological validation produced a much larger generalization gap, with mean CV MCC of 0.73 and validation MCC of 0.19; additional structural filtering reduced validation MCC to 0.15. Model-class comparisons showed substantial overlap among classifiers, and no standard algorithm consistently overcame the chronological validation penalty. These results indicate that validation-set definition has a dominant influence on estimated QSAR generalization and that chronological data set shift is not reproduced by random or structure-only validation.

## Graphical abstract

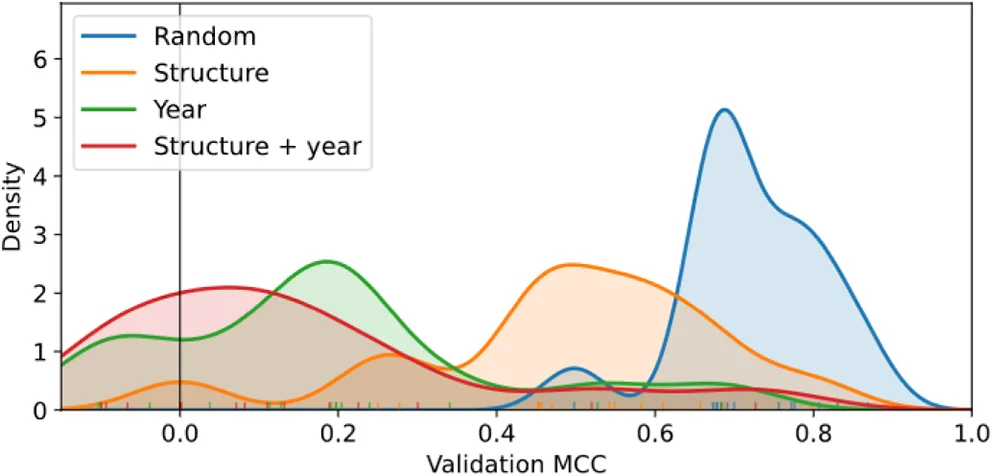

## Introduction

Quantitative structure–activity relationship (QSAR) modeling is built on the premise that chemical structure can support useful predictions of molecular properties and biological activity before experimental testing. In drug discovery, this promise is especially attractive because QSAR models can prioritize compounds, guide virtual screening, and reduce the cost of exploring chemical space. However, the central question is not whether a model can reproduce historical data, but whether it can predict compounds that were unavailable when the model was built. Validation is therefore not a secondary technical detail, but the condition under which claims of QSAR predictivity become meaningful. This principle has been emphasized repeatedly in discussions of internal and external validation, applicability domains, model development, and best-practice recommendations for machine learning in the chemical sciences.^1–4^

This issue is particularly acute in ligand-based bioactivity modeling. Public and proprietary bioactivity data sets are rarely random collections of independent molecules. They often contain analogue series, repeated measurements, evolving assay contexts, and time-dependent changes in the regions of chemical space being explored. Under these conditions, random splits and ordinary cross-validation can place close analogues in both training and validation sets, allowing models to interpolate within familiar chemical neighborhoods rather than generalize to genuinely new compounds. Thus, the central validation problem is one of independence: what does it mean for a validation compound to be truly unseen by a model trained on historical chemical data?

Several studies have shaped current understanding of this problem. Sheridan showed that random validation can be an optimistic proxy for prospective performance and argued that time-split validation better reflects the conditions under which QSAR models are used prospectively.^5^ Importantly, this work also cautioned that not all stringent splits are equally realistic, since severe leave-class-out or scaffold-style splits may create validation tasks that are harder than typical medicinal-chemistry progression. Wallach and Heifets addressed the structural side of the same problem by showing that many ligand-based classification benchmarks reward memorization rather than generalization.^6^ Their asymmetric validation embedding analysis identified train—test structural redundancy as a measurable source of inflated benchmark performance.

More recent work has further refined the relationship between structural similarity and prospective difficulty. Landrum et al. introduced SIMPD, an algorithm for generating simulated time splits that reproduce aspects of medicinal-chemistry project evolution in public data sets.^7^ Their results reinforced the view that random splits tend to overestimate performance, whereas highly stringent analogue-based splits can underestimate it. The CARA benchmark proposed by Tian and coworkers similarly emphasized that benchmark construction and splitting rules strongly influence conclusions about compound-activity prediction in realistic drug-discovery settings.^8^ Together, these studies show that random validation is often optimistic, structural redundancy can inflate performance, and time-aware evaluation is critical. What remains less directly resolved is how these explanations relate when they are tested side by side on the same data sets.

The algorithmic dimension is also relevant. QSAR modeling now commonly uses flexible machine-learning methods such as Random Forests, Support Vector Machines, gradient-boosted trees, and neural networks. Systematic algorithm comparisons have shown that model choice can matter across end points and data sets; for example, Wu and coworkers compared 16 machine-learning algorithms on 14 QSAR data sets.^9^ However, broad algorithm benchmarking answers a different question from the one addressed here. The issue is not only which model class performs best under a given benchmark, but whether any standard model class can overcome the generalization gap exposed by chronological validation. If performance decreases under chronological validation across multiple competitive classifiers, then the problem is unlikely to be solved simply by choosing a different conventional algorithm.

In this study, we ask whether retrospective QSAR optimism is mainly a structural-neighbor problem, a chronological-drift problem, or an algorithm-selection problem. Using 14 public ChEMBL bioactivity data sets, we compare five validation conditions under a controlled model-selection workflow: conventional random validation, chronological validation, size-matched random thinning, structural filtering of the random validation set, and structural filtering of the chronological validation set. Within each base partition, model development is performed on the same training cohort and the independent validation sets are used only after model selection, allowing validation-set difficulty to be examined without confounding it with changes in the training data. Four widely used QSAR model classes are evaluated—Random Forest, Support Vector Machine, XGBoost, and a feed-forward neural network—using a common circular-fingerprint representation.

The study is organized around one main hypothesis: if retrospective optimism is mainly caused by close structural analogues in the external validation set, then removing those analogues should make random validation approach chronological validation. The size-matched random-thinning control distinguishes this structural effect from the effect of simply evaluating fewer compounds. As a related question, we ask whether the chronological generalization problem is primarily algorithmic, or whether standard QSAR models fail in similar ways under chronological validation.

## Methods

### Overview of Model Development and Validation Workflow

The methodological design of this study follows the central principle that QSAR models should be selected independently from the data used for their final external evaluation. In rigorous QSAR modeling, the apparent quality of a model is meaningful only when the procedures for data curation, model optimization, model selection, and external validation are explicitly separated.^1–3^ This distinction is particularly important for ligand-based bioactivity prediction, where structurally related molecules, repeated chemical series, and temporally clustered measurements can make internal validation appear more favorable than performance on genuinely held-out compounds. Accordingly, the workflow used here was designed so that the independent validation set (IVS) remains unavailable during model building and is used only once, after the model class and hyperparameters have already been chosen.

Figure 1 summarizes the generic workflow. Two base partitions were generated for each data set: an 80/20 random partition and a chronological partition based on publication year. The training subset of each base partition was used for all model-development steps, including hyperparameter optimization and 5-fold cross-validation. After model selection, the selected models were evaluated on the IVSs from the corresponding base validation cohort. The random validation cohort was compared with a size-matched random subset and with a structurally filtered subset; the chronological validation cohort was compared with its structurally filtered counterpart. The validation metrics therefore estimate the performance of models chosen entirely from training data while progressively changing the difficulty and composition of the external validation set.

**Figure 1:**
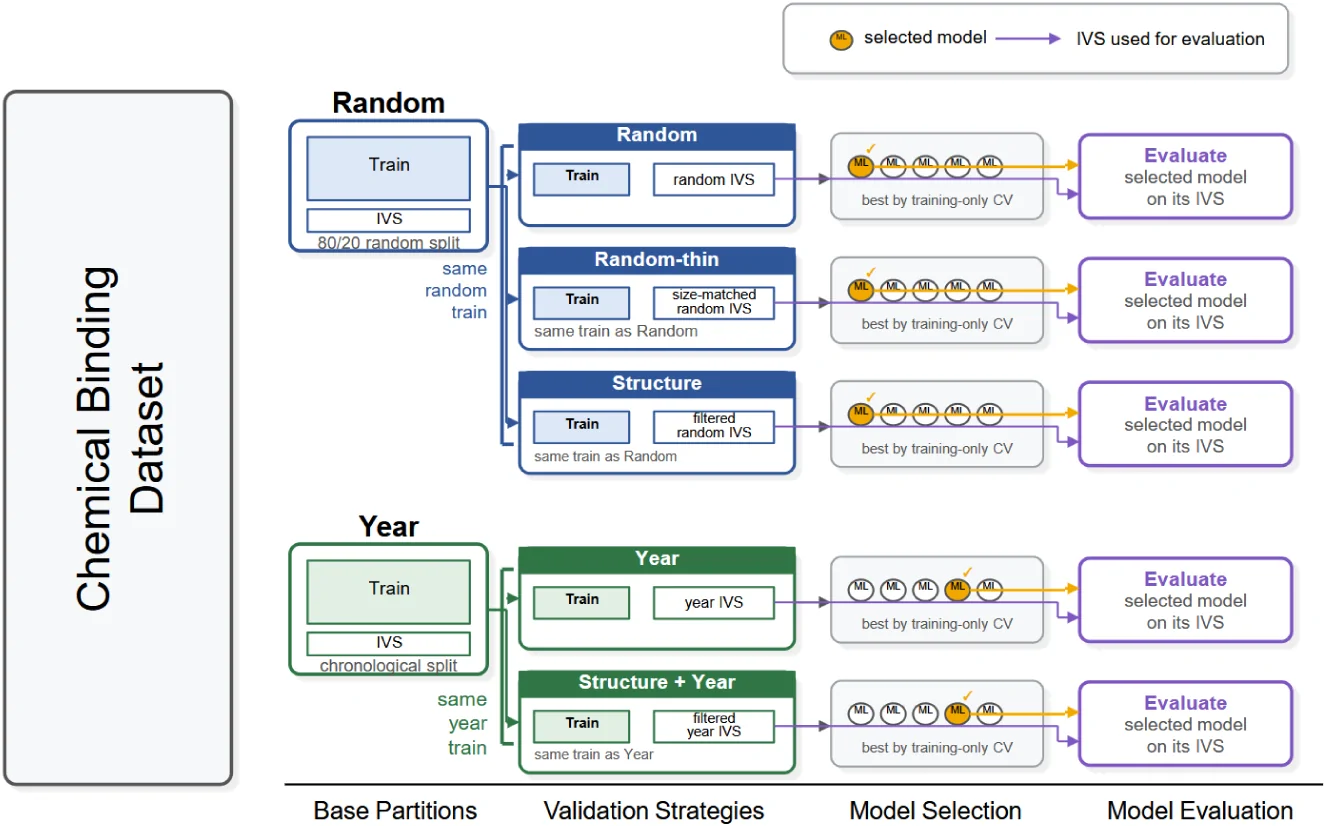
General workflow. Two base partitions, Random and Year, define the model-development cohorts. Random, Random-thin, and Structure share the same random training set but differ in their independent validation sets (IVS); Year and Structure + Year similarly share the same chronological training set. Models are selected exclusively by training-set cross-validation and subsequently evaluated on the corresponding IVS.

This design is conceptually related to best-practice QSAR workflows proposed by Tropsha and others, in which predictive modeling is treated as a complete pipeline rather than as a single fitting step.^2,10^ It also follows the logic of automated QSAR model-building frameworks, such as that of Kausar and Falcao, where candidate models are generated and compared within a controlled workflow before external validation is assessed.^11^ Here, the emphasis is not only on identifying the best-performing classifier, but on testing how the definition of the IVS changes apparent model reliability. Holding the training cohort fixed within each base partition provides a controlled comparison in which changes in external performance can be attributed to the validation cohort rather than to simultaneous changes in model-development data.

The five validation conditions were chosen to probe different sources of optimism and generalization difficulty. Random validation represents the conventional assumption that training and validation compounds are exchangeable. Year validation preserves chronological order, using earlier compounds for training and later compounds for validation.^5,7^ Structural validation removes from the random IVS compounds whose maximum Tanimoto similarity to any training compound is at or above the 0.60 cutoff, directly testing dependence on close training-set analogues.^6,10^ Random-thin retains a random subset of the same random IVS with the same number of compounds as structural validation, controlling for the reduction in validation-set size. Structure + Year applies the same structural criterion to the chronological IVS. Thus, the random, random-thin, and structure conditions share one model-development cohort, while year and structure + year share another.

All model comparisons were therefore interpreted within this framework. Internal *k*-fold cross-validation was used as the only basis for selecting among competing machine-learning models, because that is the information that would be available during model development. The IVS results were reserved for external evaluation and were not used to tune hyperparameters, select descriptors, choose a model class, or revise the partitioning.

### Molecular Representation and Descriptors

In QSAR modeling, chemical structures must be converted into numerical representations before they can be used by machine-learning algorithms. This representation is not a neutral technical detail, because it defines which structural information is available to the model and therefore constrains the type of structure–activity relationships that can be learned. Molecular descriptors range from simple physicochemical quantities, such as molecular weight or calculated lipophilicity, to graph-based descriptors, fragment counts, pharmacophore descriptors, three-dimensional descriptors, and molecular fingerprints.^3,4,12^ In the present study, all compounds were represented by extended-connectivity fingerprints with diameter six bonds (ECFP6), implemented as binary circular fingerprints with radius 3 and length 2048 bits. ECFP fingerprints are derived from the Morgan neighborhood-enumeration idea and encode atom-centered circular environments by iteratively expanding local atomic identifiers through the molecular graph.^13,14^ Thus, an ECFP6 bit vector records the presence or absence of hashed local substructural environments extending up to three bonds from each atom, providing a compact two-dimensional description of atom types, local connectivity, bond patterns, ring environments, and neighboring functional groups without requiring molecular alignment, conformer generation, target structures, or docking information.

ECFP6 was selected because circular fingerprints are a strong and widely used baseline for ligand-based QSAR and compound-activity prediction. They were developed specifically for structure–activity modeling and have become standard in similarity searching, virtual screening, and supervised bioactivity prediction.^14–16^ Their usefulness follows the neighborhood principle in medicinal chemistry: compounds sharing many local substructures often have related biological activities, although important exceptions such as activity cliffs are also well-known.^17^ Compared with simple global physicochemical descriptors, ECFP fingerprints retain more detailed information about the local chemical environments that medicinal chemists usually modify during analogue optimization. Compared with three-dimensional descriptors, they are easier to compute reproducibly from public SMILES data and avoid introducing additional variability from protonation, tautomer selection, conformer generation, or molecular alignment. A single fixed ECFP6 representation was therefore used for all data sets and model classes, so that performance differences could be attributed to validation strategy and model selection rather than to end point-specific descriptor engineering.

### Machine-Learning Models

To evaluate whether the conclusions depended on a particular learning algorithm, we considered four widely used QSAR model classes: Random Forests, Support Vector Machines, Extreme Gradient Boosting, and feed-forward neural networks. These methods represent complementary families of classifiers for molecular fingerprints—bagged decision-tree ensembles, kernel-based margin classifiers, boosted decision-tree ensembles, and neural-network classifiers. Their inclusion follows both general QSAR practice and benchmarking studies showing that no single algorithm is universally optimal across targets and data sets.^3,9,11^ Here, the goal was not to introduce a new architecture, but to test whether validation strategy remains dominant when several standard QSAR models are evaluated under the same descriptor representation and model-selection protocol.

Random Forests were used as a bagging-based tree-ensemble baseline. They combine decision trees fitted to bootstrap-resampled training data, reducing the variance of individual trees while preserving the ability to model nonlinear relationships between fingerprint bits.^18^ Their long-standing use in compound classification and QSAR makes them a natural reference method for high-dimensional and noisy molecular data.^19^

Support Vector Machines were included as kernel-based classifiers. With a radial-basis-function kernel, SVMs can learn nonlinear activity boundaries in sparse, high-dimensional fingerprint spaces by maximizing the margin between classes.^20^ This has made them a common choice in drug-discovery and QSAR applications.^21^

Extreme Gradient Boosting was used as a second tree-based strategy, distinct from Random Forests because trees are added sequentially to correct previous errors. XGBoost implements gradient boosting efficiently and includes regularization mechanisms that can improve generalization when tuned appropriately.^22^ Boosted trees have been adopted in QSAR and bioactivity prediction because they handle sparse descriptors and often perform competitively on tabular molecular data sets.^9,23^

Finally, a feed-forward neural network was included as a flexible nonlinear classifier operating on the same fixed fingerprints as the other models. Neural-network methods have been widely explored in QSAR, from shallow multilayer perceptrons to deeper representation-learning approaches.^24^ In this study, the neural network was treated as a standard classifier rather than as a separate descriptor-learning pipeline, preserving the focus on validation strategy.

### Model Quality Assessment

The predictive quality of each classifier was evaluated from the confusion matrix obtained on either the internal cross-validation folds or the independent validation set. For a binary QSAR classifier, predictions can be summarized by the number of true positives (TP), true negatives (TN), false positives (FP), and false negatives (FN). Because the data sets considered in this work are not perfectly balanced, and because bioactivity data sets often contain substantially more inactive than active compounds, model quality cannot be assessed reliably by accuracy alone. A classifier that predicts most compounds as inactive can obtain a deceptively high accuracy while failing to identify active compounds, which is precisely the type of behavior that would be undesirable in virtual screening or ligand-prioritization settings. Therefore, in line with recommended QSAR validation practice, performance was assessed using metrics that explicitly reflect both classes and the different types of classification error.^1,25^

The primary metric used in this study was the Matthews correlation coefficient (MCC). MCC is defined as eq 1

1 $$\mathrm{MCC}=\frac{TP\times TN-FP\times FN}{\sqrt{\left(TP+FP\right)\left(TP+FN\right)\left(TN+FP\right)\left(TN+FN\right)}}$$

MCC ranges from −1 to +1, where +1 represents perfect classification, 0 corresponds to no better than random association between predictions and true labels, and −1 indicates complete disagreement.^26^ Its main advantage is that it uses all four entries of the confusion matrix simultaneously. Consequently, a high MCC requires the classifier to perform well on both active and inactive compounds, while also penalizing both false positives and false negatives. This makes MCC more stringent than accuracy in imbalanced data sets: a majority-class classifier may achieve high accuracy simply by predicting the dominant class, but it will not obtain a high MCC because it fails to generate a balanced association between predicted and observed labels. MCC is also more complete than metrics focused only on the positive class, because true negatives contribute explicitly to the score.^27^

This stringency was important for the present study because the main question was not whether a model could reproduce the dominant class, but whether it could make reliable active/inactive predictions under different definitions of independence between training and validation compounds. In particular, time-split and structurally separated validation sets may change both the class balance and the chemical relationship between training and validation compounds. MCC is well suited to this setting because it remains interpretable when class proportions differ across partitions, and because it penalizes degenerate solutions such as predicting almost all compounds as inactive or almost all compounds as active. For this reason, MCC was used as the main criterion for comparing validation strategies and for selecting the best model within the cross-validation procedure.

As a complementary metric, we also report the F1 score (eq 3). Precision and recall are defined as eq 2

2 $$\mathrm{Precision}=\frac{TP}{TP+FP},\;\mathrm{Recall}=\frac{TP}{TP+FN}$$

and the F1 score is their harmonic mean:

3 $$F1=2\times \frac{\mathrm{Precision}\times \mathrm{Recall}}{\mathrm{Precision}+\mathrm{Recall}}=\frac{2TP}{2TP+FP+FN}$$

F1 is useful because it focuses directly on the quality of positive predictions: it decreases when the model misses many active compounds and also when many predicted actives are false positives. This makes it a valuable complement to MCC in QSAR classification, where the positive class is often the class of practical interest. However, F1 does not include TN and therefore cannot distinguish between models that behave very differently on the inactive class if their TP, FP, and FN counts are the same. For this reason, F1 was interpreted as a diagnostic measure of active-class recovery rather than as the primary measure of global classifier quality.^25,27^

## Data Preparation

The experimental data sets were assembled from the most current ChEMBL release (ChEMBL 37, release May 2026), a manually curated public database of bioactive molecules and their measured interactions with biological targets.^28,29^ Fourteen human protein targets were resolved from their UniProt accessions to ChEMBL single-protein targets and the corresponding IC_50_, K_*i*_, or EC_50_ activity records were downloaded programmatically together with structures and publication-year metadata. No assay-confidence filtering was applied at retrieval; activity relations, units, structures, and publication-year eligibility were handled subsequently during curation. The resolved ChEMBL target identifiers and retrieval metadata are provided in the Supporting Information. The selected targets include kinases, G-protein-coupled receptors, transporters, enzymes, and nuclear receptors, thereby reducing the likelihood that the conclusions reflect the behavior of a single target family.

For each target, activity records were retrieved programmatically from ChEMBL for the corresponding target and selected end point. The activity end point used for each data set was selected from the dominant binding or functional measurement available for that target, namely IC_50_, K_*i*_, or EC_50_. Each data set was then converted into a binary classification problem in which compounds were labeled as active or inactive according to a stringent potency threshold of 100 nM. This threshold was chosen deliberately. The goal of the study was not to construct optimally balanced target-specific QSAR models, but to evaluate validation strategies under conditions closer to ligand prioritization in drug discovery, where strongly active compounds are expected to be less frequent than inactive or weakly active compounds. By adopting a stringent definition of activity, the benchmark was intentionally biased toward negative examples rather than toward artificially balanced classification tasks. The common 100 nM cutoff was used as a benchmarking convention and does not imply mechanistic equivalence between IC_50_, K_*i*_, and EC_50_ measurements.

Table 1 summarizes the data sets before and after curation. Across the 14 targets, 78,893 activity records were retrieved and 43,573 compounds were retained after preprocessing and label assignment. The curated data sets contained 26,590 inactive and 16,983 active compounds, corresponding to an overall positive fraction of 39.0%. Most data sets remained negative-majority, although CDK4 contained 70.7% actives and IGF1R was exactly balanced. The target-to-target variation was retained rather than imposing an artificial common class balance.

**Table 1:** Description of the ChEMBL Target Data Sets Used in This Study^a^

Gene	Target protein	Activity	Retrieved records	Curated records	Negatives	Positives	Active (%)
EGFR	Epidermal growth factor receptor	IC50	26600	11908	6463	5445	45.7
DRD2	D(2) dopamine receptor	*K*_i_	14842	9480	6469	3011	31.8
ERBB2	Receptor tyrosine-protein kinase erbB-2	IC50	5265	3111	1642	1469	47.2
DAT	Sodium-dependent dopamine transporter	*K*_i_	3467	1941	1231	710	36.6
HRH1	Histamine H1 receptor	*K*_i_	2893	1598	995	603	37.7
IGF1R	Insulin-like growth factor 1 receptor	IC50	4604	2736	1368	1368	50.0
IKBKB	Inhibitor of nuclear factor kappa-B kinase subunit beta	IC50	2336	1289	941	348	27.0
TNF	Tumor necrosis factor	IC50	1616	939	784	155	16.5
CDK4	Cyclin-dependent kinase 4	IC50	1787	1456	426	1030	70.7
HTR1B	5-hydroxytryptamine receptor 1B	*K*_i_	1328	1075	603	472	43.9
MAOA	Amine oxidase [flavin-containing] A	*K*_i_	1865	768	659	109	14.2
ADRB1	Beta-1 adrenergic receptor	IC50	1547	637	550	87	13.7
PPARG	Peroxisome proliferator-activated receptor gamma	EC50	5448	4022	3145	877	21.8
ABL1	Tyrosine-protein kinase ABL1	IC50	5295	2613	1314	1299	49.7

a Retrieved records correspond to the initial number of activity records obtained from ChEMBL, whereas curated records correspond to the final number of compounds retained after preprocessing and activity-label assignment.

### Data Processing and Curation

The raw ChEMBL activity records were processed to obtain one standardized binary classification data set per target. Records lacking the information required for modeling, including a valid molecular identifier, structure, activity value, unit, activity relation, or publication year, were removed. Molecular structures were represented by SMILES and validated before descriptor calculation. When multiple activity records were available for the same compound, records were classified independently and compounds with conflicting active/inactive assignments were excluded. Otherwise, a single consensus activity label was retained. For chronological partitioning, temporal availability was defined by the earliest documented publication year for each compound.

Activity values were converted to nM and classified using a 100 nM threshold, with values at or below 100 nM considered active. Censored measurements were retained only when their reported bound determined the class unambiguously: upper-bound values were assigned active only when the bound was at or below 100 nM, and lower-bound values were assigned inactive only when the bound excluded activity at the threshold. Ambiguous censored records were excluded. Across the 14 data sets, 1,197 censored records (about 1.5% of the total) were excluded because their reported bounds did not determine the activity class unambiguously at the 100 nM threshold.

The resulting curated data sets contained a compound identifier, validated molecular structure, binary activity label, and first documented publication year. Activity labels were used for model training and evaluation, whereas publication year was used only to define chronological partitions and was not included as a model descriptor.

### Data Set Partitioning and Validation-Set Composition

After curation and activity-label assignment, two base train-validation partitions were generated. The random strategy used an 80/20 random split with a fixed seed. The year strategy sorted compounds by ChEMBL document year and assigned the most recent compounds to the IVS, with compounds from the cutoff year kept together; its final validation fraction could therefore differ from exactly 20%. Structural filtering was then applied only to the corresponding IVS, leaving its training cohort unchanged. For each validation compound, the maximum Tanimoto similarity between its ECFP6 fingerprint and any training compound was calculated, and compounds at or above the 0.60 similarity cutoff were excluded. This cutoff value was selected as a deliberately conservative criterion for removing structurally related validation compounds; similarity thresholds are fingerprint- and data set-dependent, and the aim was to impose substantial structural separation rather than to define a universal boundary of molecular similarity.^30^ This generated the Structure IVS from the random partition and the Structure + Year IVS from the chronological partition. A Random-thin control was additionally generated by randomly sampling, from the original random IVS, the same number of compounds retained by structural filtering. This permits the loss caused by structural novelty to be distinguished from any effect caused simply by reducing the number of validation compounds.

The actual positive/negative balance was recorded for every training and validation cohort (Table 2). Because structural filtering and random thinning modify only the IVS, Random, Random-thin, and Structure have identical training cohorts, while Year and Structure + Year likewise share their training cohort. Differences in their external performance therefore reflect validation-set composition rather than retraining on different samples. The year-derived partitions may additionally introduce shifts in class prevalence, making them a more realistic but also more demanding approximation of future-use QSAR prediction.

**Table 2:** Class Composition of the Training and Validation Sets for Each Data Set and Validation Strategy^a^

	Random		Year		Random-thin		Structure		Structure + Year	
Data set	Train	Validation	Train	Validation	Train	Validation	Train	Validation	Train	Validation
EGFR	4362/9526	1083/2382	3631/8998	1814/2910	4362/9526	141/311	4362/9526	54/311	3631/8998	1617/2590
DRD2	2390/7584	621/1896	2575/7581	436/1899	2390/7584	69/234	2390/7584	52/234	2575/7581	380/1663
ERBB2	1175/2488	294/623	979/2381	490/730	1175/2488	34/74	1175/2488	11/74	979/2381	442/665
DAT	567/1552	143/389	549/1530	161/411	567/1552	29/94	567/1552	22/94	549/1530	145/384
HRH1	490/1278	113/320	512/1246	91/352	490/1278	22/64	490/1278	17/64	512/1246	63/318
IGF1R	1095/2188	273/548	726/1766	642/970	1095/2188	32/78	1095/2188	7/78	726/1766	639/962
IKBKB	272/1031	76/258	297/999	51/290	272/1031	17/56	272/1031	5/56	297/999	38/239
TNF	122/751	33/188	92/723	63/216	122/751	5/20	122/751	4/20	92/723	63/216
CDK4	828/1164	202/292	687/1053	343/403	828/1164	24/36	828/1164	11/36	687/1053	315/367
HTR1B	375/860	97/215	454/859	18/216	375/860	24/39	375/860	6/39	454/859	13/206
MAOA	90/614	19/154	49/603	60/165	90/614	3/38	90/614	7/38	49/603	56/145
ADRB1	69/509	18/128	20/452	67/185	69/509	1/15	69/509	3/15	20/452	62/178
PPARG	696/3217	181/805	753/3103	124/919	696/3217	17/71	696/3217	9/71	753/3103	118/839
ABL1	1004/2090	295/523	888/1953	411/660	1004/2090	42/75	1004/2090	18/75	888/1953	367/576

a Values are reported as positive compounds/total compounds; the number of negative compounds is obtained as total minus positives.

Nearest-training-neighbor similarities showed the expected separation between validation conditions. Across the 14 data sets, the median of the target-level median ECFP6 Tanimoto similarities was 0.750 for Random, 0.753 for Random-thin, 0.465 for Structure, 0.287 for Year, and 0.280 for Structure + Year; target-level median and interquartile-range values are provided in the Supporting Information.

## Model Implementation and Hyperparameter Selection

All machine-learning experiments were implemented as a controlled model-selection pipeline applied independently to each data set and validation strategy. Molecular descriptors were fixed before model fitting, and all classifiers received the same binary ECFP6 fingerprint representation. Labels were encoded as 1 for active compounds and 0 for inactive compounds.

The four model classes were implemented using standard open-source machine-learning libraries. Random Forests and Support Vector Machines were implemented with scikit-learn.^31^ Extreme Gradient Boosting models were implemented with XGBoost.^22^ Feed-forward neural networks were implemented in PyTorch^32^ and optimized with Adam.^33^ These four model classes were chosen because they represent complementary families commonly used in ligand-based QSAR modeling: bagged tree ensembles, kernel-based classifiers, boosted tree ensembles, and neural-network classifiers.^9,18,20,22,24^

For each data set, validation strategy, and model class, hyperparameters were selected exclusively by internal cross-validation on the training set. We used stratified 5-fold cross-validation with shuffled folds and a fixed random seed. Each candidate hyperparameter configuration was fitted on four folds and evaluated on the remaining fold; this procedure was repeated for all five folds. The primary model-selection criterion was the mean cross-validated Matthews correlation coefficient (MCC), although the implementation also recorded accuracy, balanced accuracy, precision, recall, specificity, negative predictive value, F1 score, ROC-AUC, and average precision. After the best hyperparameter setting had been selected within a model class, the corresponding model was refitted on the complete training set and evaluated once on the independent validation set. The independent validation set was not used for hyperparameter tuning, model-class selection, threshold adjustment, or any other model-development decision.

Table 3 summarizes the hyperparameter grids used in the experiments. Each of the four model classes was evaluated over 18 candidate configurations, giving 72 configurations and 360 internal 5-fold cross-validation fits per data set and validation condition. Across 14 data sets and five validation conditions, the complete benchmark comprises 25,200 internal cross-validation fits and 280 final model-class refits. Because the random, random-thin, and structure conditions share a training cohort, as do year and structure + year, their identical training-only CV results are expected by design.

**Table 3:** Hyperparameter Grids Used for Model Selection^a^

Model class	Implementation	Hyperparameters searched	No. configurations
Random Forest	scikit-learn	class_weight: {balanced_subsample}	18
		max_depth: {None}	
		max_features: {0.3, sqrt}	
		min_samples_leaf: {1, 3, 5}	
		n_estimators: {200, 400, 800}	
Support Vector Machine	scikit-learn	C: {0.5, 1, 2, 10, 50, 100}	18
		class_weight: {balanced}	
		gamma: {0.001, 0.01, scale}	
		kernel: {rbf}	
XGBoost	XGBoost	colsample_bytree: {0.8}	18
		learning_rate: {0.03, 0.1}	
		max_depth: {3, 6, 9}	
		n_estimators: {200, 500, 800}	
		reg_lambda: {1}	
		subsample: {0.8}	
		use_scale_pos_weight: {True}	
Neural Network	PyTorch	batch_size: {64}	18
		dropout: {0.1, 0.25}	
		early_stopping_fraction: {0.1}	
		hidden_layers: {(256), (512, 256, 64), (512, 64)}	
		learning_rate: {1e-04, 3e-04, 0.001}	
		max_epochs: {150}	
		patience: {12}	
		use_pos_weight: {True}	
		weight_decay: {1e-05}	

a The values were reconstructed from the complete hyperparameter cross-validation result files generated by the modeling runs.

The Random Forest classifier was used as a bagging-based tree ensemble. Each configuration used class-balanced bootstrap weighting through balanced_subsample, allowing class imbalance to influence tree construction while preserving the same global decision threshold across data sets. The Support Vector Machine used a radial-basis-function kernel and class-balanced weighting. Because probabilistic calibration was not required for the primary operating-point metrics, probability estimation was disabled; continuous scores for ranking metrics were obtained from the decision function.

The XGBoost classifier used the binary logistic objective and log-loss as the internal optimization criterion. Histogram-based tree construction was used for computational efficiency. The searched grid varied the number of boosting rounds, tree depth, and learning rate, while subsampling, column subsampling, and L2 regularization were kept fixed to provide a moderate degree of regularization across all data sets.

The neural-network model was a fully connected multilayer perceptron operating directly on the ECFP6 fingerprint vector. Each hidden layer was followed by a ReLU activation and dropout, and the final layer produced a single logit trained with binary cross-entropy with logits. Class imbalance was handled by weighting the positive class according to the inactive-to-active ratio in the corresponding fitting data. Candidate networks were trained for up to 150 epochs; an internal 10% training-only holdout with patience 12 selected the epoch count, after which the network was refitted from scratch on all fitting samples for that number of epochs. Adam optimization and a 0.5 classification threshold were used. No IVS information was used for early stopping or model selection.

For reproducibility, the implementation used a fixed default random seed for NumPy, Python, and PyTorch. Cross-validation folds were generated using stratified splitting, ensuring that each fold approximately preserved the active/inactive ratio of the training data.

The full source code and data are made available in a Zenodo archive.

## Results

Figure 2 provides an overview of the principal validation results across the 14 data sets. It contrasts internal 5-fold cross-validation performance with independent validation performance under the main validation conditions, highlighting the close agreement observed for Random validation and the progressive deterioration associated with structural and especially chronological separation (Panels A and B). The density plots further summarize the shift in the overall distribution of MCC values across validation strategies (Panels C and D). Complementary Table 4 summarizes performance under the five validation conditions. For each data set, the reported validation score corresponds to the model selected by the highest training-only 5-fold cross-validation MCC. Random, Random-thin, and Structure therefore share the same CV estimates and selected model for each data set, as do Year and Structure + Year; only the external validation cohort differs within each group.

**Figure 2:**
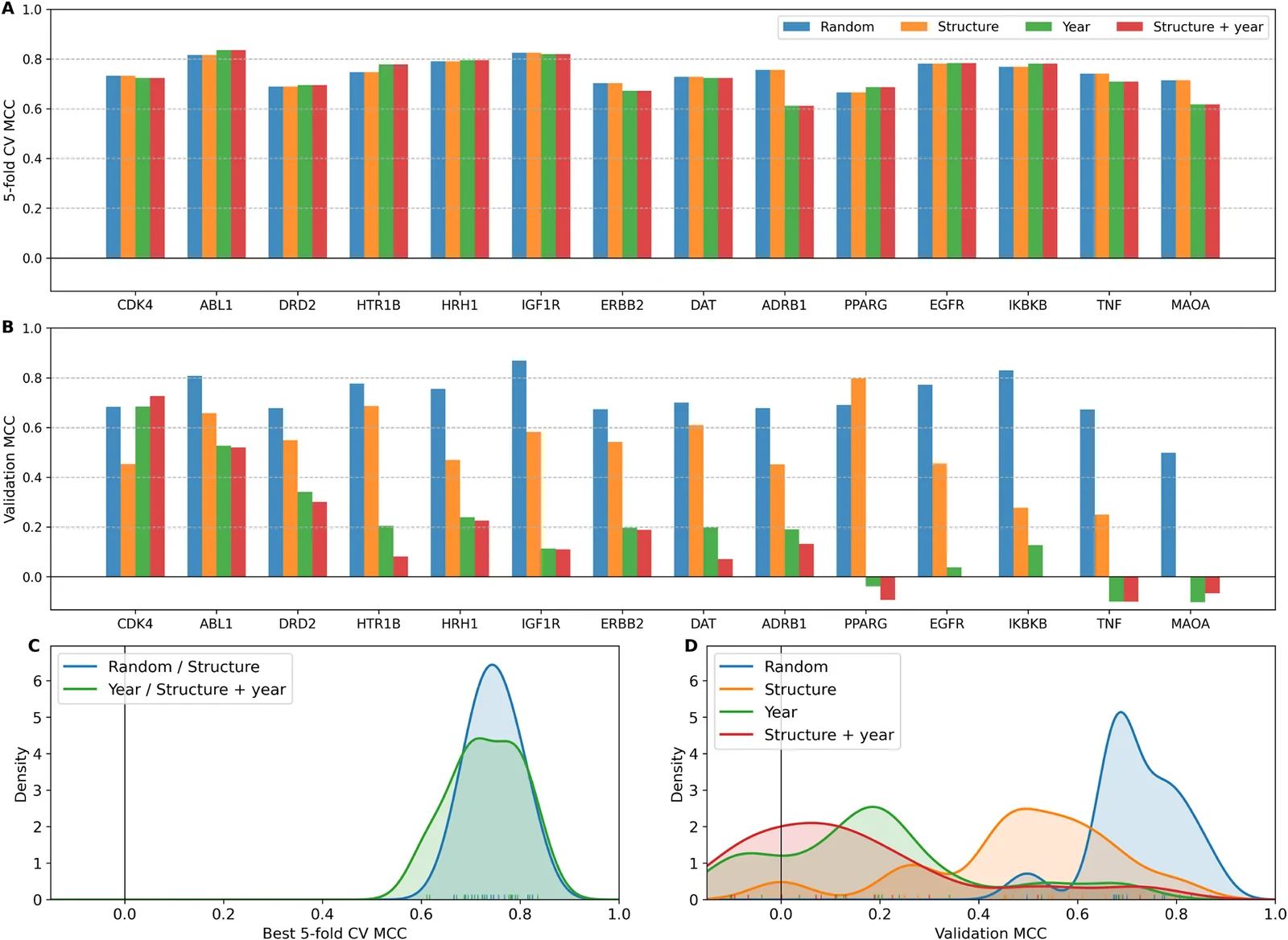
Main results. Panel A: 5-fold cross-validation MCC for the 14 data sets under the four principal validation conditions. Random and Structure share identical training-only cross-validation results, as do Year and Structure + Year, because structural filtering is applied only to the external validation cohort. Panel B: independent validation-set MCC for the same four conditions, showing the divergence in performance produced by increasingly demanding validation cohorts. Panel C: density distribution of 5-fold cross-validation MCC; because the paired conditions share identical training sets, the coincident distributions are shown jointly as Random/Structure and Year/Structure + Year. Panel D: density distribution of independent validation-set MCC for the four validation conditions. The Random-thin size-matched control is reported separately in Tables 2, 4, and 5.

**Table 4:** Average MCC and F1 Performance of the Best Model Found for Each Data Set According to Validation Strategy^a^

Validation strategy	5-fold CV MCC	Validation MCC	5-fold CV F1	Validation F1
Random	0.75 (0.05)	0.72 (0.09)	0.83 (0.06)	0.82 (0.10)
Year	0.73 (0.07)	0.19 (0.22)	0.81 (0.09)	0.34 (0.28)
Random-thin	0.75 (0.05)	0.73 (0.09)	0.83 (0.06)	0.82 (0.11)
Structure	0.75 (0.05)	0.48 (0.20)	0.83 (0.06)	0.55 (0.21)
Structure + Year	0.73 (0.07)	0.15 (0.24)	0.81 (0.09)	0.29 (0.29)

a Values are mean scores with sample standard deviation in parentheses across data sets. For each data set and validation strategy, the model was selected according to the highest 5-fold CV MCC.

The validation condition had a strong effect on apparent model reliability. Random validation produced closely aligned internal and external estimates (MCC 0.75 versus 0.72), and reducing the random IVS to the same size as the structurally filtered IVS did not reduce performance (validation MCC 0.73). In contrast, structural filtering of that size-matched external cohort reduced validation MCC to 0.48 while leaving the training-only CV estimate unchanged at 0.75. Chronological validation produced a much larger mismatch, with CV MCC 0.73 but validation MCC only 0.19; additional structural filtering of the chronological IVS yielded a mean MCC of 0.15. Thus, the structural penalty cannot be attributed simply to smaller validation sets, while chronological separation remains substantially more demanding than structural novelty alone.

The aggregate results indicate that the training cohorts retain substantial learnable signal, whereas external performance depends strongly on how independence is defined. Random and Random-thin validation remain closely aligned with internal CV, structural filtering shifts validation performance downward, and the year-derived conditions show the largest generalization gaps.

To test whether these differences were systematic across targets, validation MCC values were compared pairwise using each data set as a paired block. Six planned comparisons were used to separate the main effects examined in the present design: the effect of reducing validation-set size (Random versus Random-thin), the effect of structural filtering at matched validation-set size (Random-thin versus Structure), the overall structural penalty relative to ordinary random validation, the chronological penalty relative to random validation, the difference between structural and chronological separation, and the additional effect of structural filtering within the chronological validation set. Results are summarized in Table 5.

**Table 5:** Paired Comparisons of Validation MCC between Validation Conditions^a^

Comparison	n	Mean ΔMCC	Median ΔMCC	95% CI	*p*_adj_ Wilcoxon
Random – Random-thin	14	–0.008	–0.005	[−0.055, 0.035]	1.0000
Random-thin – Structure	14	0.244	0.217	[0.142, 0.357]	0.0017
Random – Structure	14	0.236	0.228	[0.148, 0.325]	0.0017
Random – Year	14	0.533	0.544	[0.417, 0.638]	0.0015
Structure – Year	14	0.297	0.303	[0.174, 0.419]	0.0026
Year – Structure + Year	14	0.037	0.025	[0.010, 0.065]	0.0332

a Differences were calculated data set by data set as the MCC of the first condition minus the MCC of the second condition. Positive values indicate higher validation MCC for the first condition. Confidence intervals are bootstrap 95% confidence intervals for the paired mean difference across data sets. Statistical significance was assessed with a two-sided paired Wilcoxon signed-rank test; adjusted *p* values were obtained using the Benjamini–Hochberg procedure across the six planned comparisons.

The paired tests confirm and sharpen the main interpretation. Random thinning alone had no systematic effect on validation performance (mean ΔMCC = −0.008, adjusted *p* = 1.000), whereas replacing the size-matched random subset with structurally filtered compounds produced a substantial decrease (Random-thin – Structure mean ΔMCC = 0.244, 95% CI [0.142, 0.357], adjusted *p* = 0.0017). The corresponding Random – Structure comparison was similarly significant (mean ΔMCC = 0.236, adjusted *p* = 0.0017). Thus, the structural penalty is attributable to validation-set composition rather than simply to the smaller number of validation compounds.

Chronological validation produced the largest loss relative to ordinary random validation (mean ΔMCC = 0.533, 95% CI [0.417, 0.638], adjusted *p* = 0.0015) and remained significantly more difficult than structure-only validation (Structure – Year mean ΔMCC = 0.297, adjusted *p* = 0.0026). This difference indicates that structural novelty alone does not account for the chronological generalization penalty.

Structural filtering within the chronological validation set produced a small but systematic additional decrease when structural filtering is imposed within the chronological validation set. Year validation exceeded Structure + Year by a mean MCC of 0.037 (95% CI [0.010, 0.065], adjusted *p* = 0.0332). The magnitude of this effect is modest compared with the random-to-year or structure-to-year differences, but it indicates that structural novelty retains an additional measurable contribution even after chronological separation.

Taken together, the paired analysis supports two principal conclusions. First, the loss produced by structural filtering reflects the composition of the validation cohort rather than merely its reduced size, because the size-matched Random-thin control retains random-like performance. Second, chronological validation reveals a substantially more demanding generalization problem than structural separation alone. The small additional penalty observed for Structure + Year further indicates that temporal and structural shifts are related but not interchangeable sources of generalization difficulty.

A leave-one-target-out sensitivity analysis showed that this chronological penalty was not driven by any individual data set: after omitting each target in turn, the mean Random–Year MCC difference remained between 0.515 and 0.574, with the paired Wilcoxon test remaining significant in every case (*p* ≤ 0.00049)

Also as a threshold-independent sensitivity analysis, ROC-AUC showed the same deterioration under chronological validation, decreasing from a mean of 0.934 under Random validation to 0.698 under Year validation (mean paired difference = 0.237, 95% CI [0.176, 0.302]; paired Wilcoxon *p* = 0.00024). Across the 14 targets, the magnitude of the change in validation-set active-class prevalence was not associated with the corresponding MCC loss (Spearman ρ = 0.134, *p* = 0.648). Thus, the chronological performance decrease cannot be attributed simply to changes in class prevalence.

The full results, including accuracy, balanced accuracy, precision, recall, specificity, negative predictive value, ROC-AUC, average precision, and all tested hyperparameter configurations, are provided as Supporting Information.

### Analysis of Top-Performing Models

Because model selection was performed exclusively by training-set cross-validation, only two distinct model-selection regimes exist in this design. Random, Random-thin, and Structure share the same random training cohort, whereas Year and Structure + Year share the same chronological training cohort. For the random-derived training sets, SVM was selected in 9 of 14 data sets, XGBoost in 4, and Random Forest in 1; for the year-derived training sets, SVM was selected in 7 data sets, XGBoost in 5, Random Forest in 1, and the Neural Network in 1 (Table 6). Thus, changes in external performance across paired validation conditions cannot be attributed to different model-class selections, reinforcing the conclusion that the dominant effect arises from the validation distribution rather than from algorithm choice.

**Table 6:** Frequency with Which Each Machine-Learning Model Class Was Selected as the Best-Performing Model by Internal 5-Fold Cross-Validation MCC under the Two Distinct Model-Development Cohorts^a^

Model class	Random-derived training cohort	Year-derived training cohort
RF	1	1
SVM	9	7
XGBoost	4	5
NN	0	1
Total	14	14

a Random, Random-thin, and Structure share the same random training cohort and therefore the same CV-based model selection; Year and Structure + Year likewise share the same chronological training cohort. RF: Random Forest; SVM: Support Vector Machine; NN: Neural Network.

### Model-Class Performance Analysis

A model-class assessment provides a complementary view to the analysis based only on the top-selected model per data set. For each of the four model families, the hyperparameter configuration was selected independently using training-only 5-fold cross-validation before evaluation on the corresponding external validation set. This allows the effect of validation design to be compared after each model family has already received its own CV-based optimization. Across model classes, the resulting validation MCC distributions were broadly similar within each validation condition, with substantial overlap between Random Forest, SVM, XGBoost and Neural Network, despite SVM being selected most frequently by internal cross-validation. In contrast, the shift between validation conditions was pronounced: all four model families performed well under Random validation, declined under structural separation, and showed substantially lower performance under Year and Structure + Year validation. Thus, no model family consistently avoided the deterioration associated with increasingly demanding external validation cohorts. Figure 3 compares the validation-set MCC distributions of the four model classes across the principal validation conditions, providing a visual assessment of whether any model family consistently mitigates the loss associated with structural or chronological separation.

**Figure 3:**
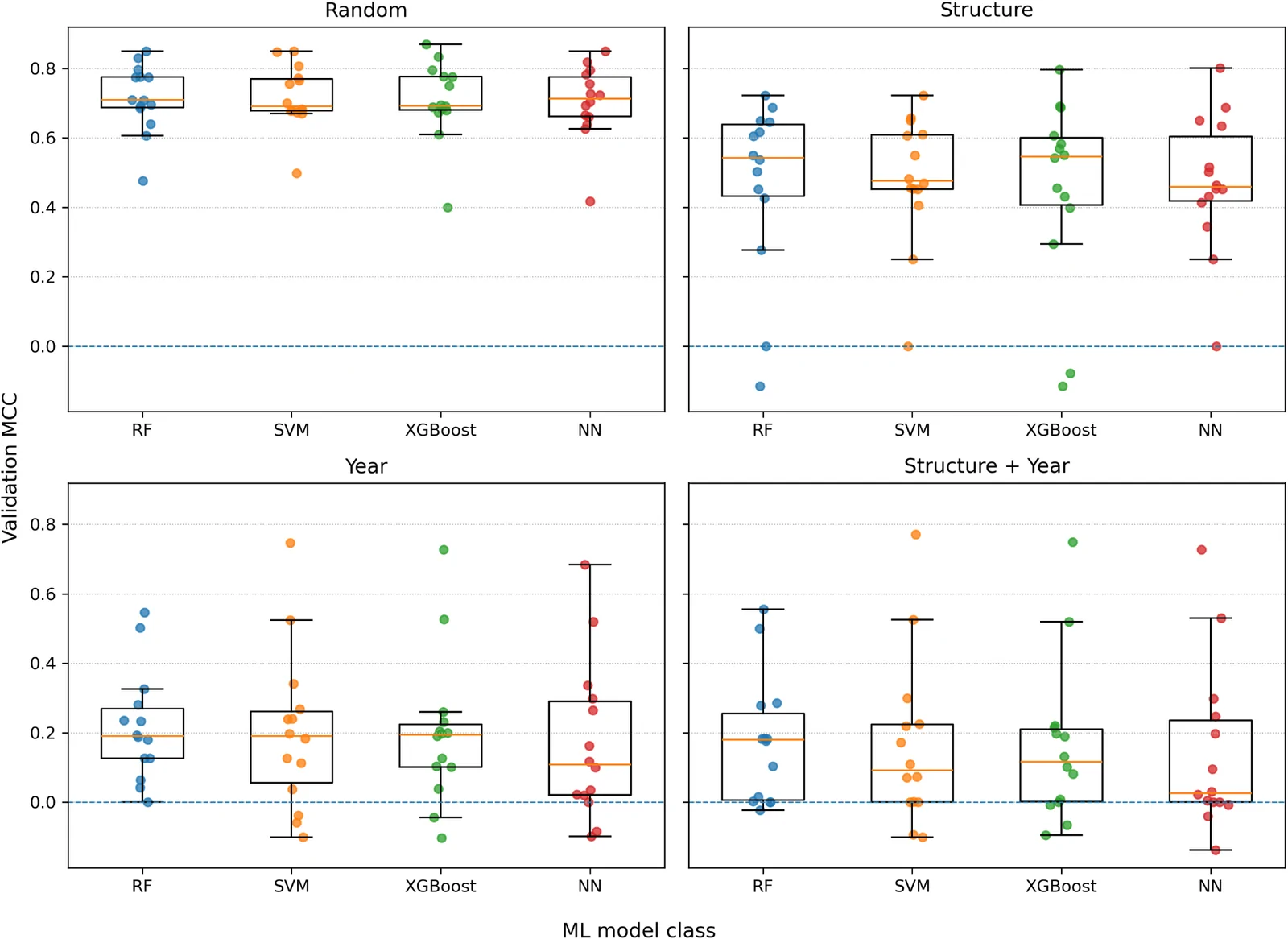
Validation-set MCC distributions by machine-learning model class across the four principal validation conditions. For each data set, validation strategy and model class, the hyperparameter setting selected was the one with the highest 5-fold cross-validation MCC on the training set. Each boxplot therefore contains 14 values, one per data set, and individual data set-level results are shown as points.

These results reinforce the conclusion that validation strategy exerts a larger influence on estimated generalization performance than the choice among the conventional model classes examined here. Hyperparameter optimization and model-family selection can affect performance within a given validation setting, but these differences are small relative to the systematic loss observed when structural or chronological distribution shift is introduced. The practical implication is that improving QSAR reliability depends primarily on validation designs that expose such shifts rather than on replacing one conventional classifier with another.

## Discussion and Conclusions

This study addresses a practical question at the center of QSAR validation: whether strong retrospective performance can be taken as evidence of prospective usefulness. The results indicate that it cannot, at least not without additional qualification. Models selected by internal cross-validation behaved reliably under random validation, but this apparent reliability did not transfer to chronological validation. This finding reinforces the view that QSAR validation should be judged by how closely the validation design matches the intended use of the model, not only by the numerical strength of internal performance estimates.^5,7^

The main contribution of this work is to separate three explanations that are often discussed together: structural similarity between training and validation compounds, temporal data set evolution, and algorithm choice. In this study, structural filtering is applied only to the external validation cohort while the model-development cohort is held fixed. The size-matched Random-thin control shows that the resulting loss is not explained merely by evaluating fewer compounds. Structural novelty therefore contributes substantially to retrospective optimism, but it does not reproduce the much larger loss observed under chronological validation. Moreover, Structure + Year validation produced a small but significant additional decrease relative to Year alone, indicating that structural and temporal shifts are related but not interchangeable sources of generalization difficulty.

The comparison across model classes leads to a second important conclusion. Random Forests, Support Vector Machines, XGBoost, and feed-forward neural networks all provided plausible QSAR classifiers after independent hyperparameter optimization by training-only cross-validation. Within each validation condition, their external MCC distributions were broadly similar and strongly overlapping, whereas the shift between validation conditions was much larger. Thus, no model family consistently avoided the performance loss associated with structural or chronological distribution shift. This does not imply that algorithm choice is irrelevant; rather, it shows that choosing among standard supervised classifiers is unlikely to compensate for an unrealistically easy validation design.

These findings have direct implications for QSAR benchmarking. Random cross-validation remains useful for model development, hyperparameter selection, and confirming that a data set contains learnable signal. Structurally filtered validation is also valuable because it tests dependence on close analogues. Nevertheless, neither random nor structure-only validation should be treated as a substitute for chronological evaluation when the goal is to estimate future predictive utility. Whenever temporal metadata are available, time-aware validation should be reported alongside conventional internal validation, and the class balance and structural relationship between training and validation sets should be made explicit.

Several limitations should be considered. Publication year is only a proxy for the true time at which compounds became available in a discovery project. The conclusions regarding model choice are restricted to the four evaluated model families—Random Forest, Support Vector Machine, XGBoost, and feed-forward neural networks—under the fixed ECFP6 representation used throughout the study. These are established and competitive QSAR approaches that achieve state-of-the-art or near-state-of-the-art performance on many conventional QSAR benchmarks, but the present results should not be generalized to all possible molecular representations or learning architectures. Structural filtering represents one operational definition of external analogue novelty and, because the training cohort is deliberately retained, it does not test the effect of rebuilding models on a globally redundancy-reduced data set. The Random-thin control addresses the effect of validation-set size, but other aspects of validation-set composition may also influence performance. Finally, despite the stringent activity threshold, public ChEMBL data sets remain enriched relative to many real screening settings. Future studies should examine other structural definitions, lower active prevalences, and genuinely prospective experimental test sets.

In conclusion, this work shows that retrospective QSAR optimism is not explained by a single factor. Structural similarity between training and validation compounds contributes to inflated random-split performance, but chronological validation exposes an additional and substantially more demanding form of generalization failure. Structural novelty retains a smaller additional effect even within chronological validation, and standard machine-learning classifiers do not eliminate these gaps. The central message is therefore methodological: QSAR models intended for prospective use should be evaluated with validation designs that approximate their intended deployment conditions. Otherwise, high cross-validation performance may measure interpolation within historical chemical space rather than predictive value for future molecular discovery.

## Supplementary Material



## Data Availability

The code and data supporting this study are available from Zenodo at 10.5281/zenodo.21936215. The archive includes the source code used for data set construction and model fitting, the curated data sets, and the data used to generate the validation splits and reported results.

## Notes

Results for all validation strategies, model classes, and data sets; data flow information and the ChEMBL manifest; and the best model selected for each data set under each validation condition (ZIP)
